# Positive Outcomes of Supplementation with Lecithin-Based Delivery Formulation of *Curcuma longa* and *Boswellia serrata* in IBS Subjects with Small Bowel Dysbiosis

**DOI:** 10.3390/life14111410

**Published:** 2024-11-01

**Authors:** Attilio Giacosa, Gaetan Claude Barrile, Simone Perna, Mariangela Rondanelli

**Affiliations:** 1 Italian Diagnostic Center (CDI), 20147 Milan, Italy; attilio.giacosa@gmail.com; 2 Department of Public Health, Experimental and Forensic Medicine, University of Pavia, 27100 Pavia, Italy; mariangela.rondanelli@unipv.it; 3 Department of Food, Environmental and Nutritional Sciences, Division of Human Nutrition, University of Milan, 20133 Milan, Italy; simoneperna@hotmail.it

**Keywords:** bloating, dysbiosis, *Boswellia serrata*, *Curcuma longa*, irritable bowel syndrome, microbiota, phytosome

## Abstract

Background: Small bowel dysbiosis (SBD) is a frequent finding in subjects with irritable bowel syndrome (IBS). The sunflower lecithin (phytosome) formulation of *Curcuma longa* and *Boswellia serrata* demonstrated beneficial effects on intestinal microbiota. This study aimed to evaluate the impact of a lecithin-based delivery formulation of *Curcuma longa* and *Boswellia serrata* extracts (CUBO) on SBD in IBS subjects. Subjects: Forty-nine adult subjects with IBS and SBD were randomly supplemented for 30 days with CUBO and a low-FODMAP diet (LFD) (intervention) or with LFD and placebo (control group). Results: The intervention group showed a significant reduction in urinary indican (*p* < 0.001), which was the marker of SBD, and of abdominal bloating (*p* < 0.001) and abdominal pain (*p* < 0.001). The pre–post control group analysis did not evidence significant differences. The comparison between the two groups (net effect in intervention minus control subjects) showed that the changes differ significantly for urinary indican *p* < 0.001 (−42.88; 95% CI: −62.04 to −23.72), abdominal bloating *p* < 0.001 (−1.50; 95% CI: −1.93 to −1.07), and abdominal pain *p* < 0.001 (−2.37; 95% CI: −3.61 to −1.13) and for the global assessment of efficacy (*p* < 0.001). The efficacy was 20% greater in males than in females. Conclusions: In IBS subjects, the intervention with CUBO and LFD shows a significantly higher reduction in SBD, abdominal pain, and bloating compared to LFD and placebo. Additional research is needed to confirm these data.

## 1. Introduction

Small bowel dysbiosis (SBD) is an abnormal change in the overall bacterial population in the small bowel with bacterial increase and the presence of types of bacteria not commonly found in the small intestine. Growing knowledge suggests that disorders of the gut microbiota are involved in the pathophysiology of irritable bowel syndrome (IBS). This is based on alterations of brain–gut axis interaction [1,2,3]. The optimal functionality of the intestinal microbiota relies on delicate balances threatened by stress, poor diet, inflammation, and illnesses. An imbalance in the intestinal microbiota, that is, gut microbiome dysbiosis and, more specifically, SBD, is a frequent finding in subjects with irritable bowel syndrome (IBS) [4].

In IBS patients, SBD may be responsible for motility alterations, increased intestinal permeability, decreased absorption of bile salts, gut inflammation, and altered enteral and central neuronal activity with clinical evidence of abdominal pain, abdominal distention, and changes in bowel habits [4]. The treatment of SBD is a matter of debate, and there is no scientific evidence on which approach is best for treating SBD. A low-FODMAP diet is a well-recognized active treatment of IBS, and it is efficacious for symptom relief in these patients [5,6]. The antioxidant and anti-inflammatory effect of curcumin and *Boswellia serrata* extracts and their beneficial modulation of intestinal microbiota suggested their potential role in IBS treatment and their potential synergism [7]. A random-effects meta-analysis published in 2018 showed that curcumin treatment is safe and that it may relieve IBS symptoms without relevant side effects [7]. Curcumin or turmeric extract helps improve IBS with a significant reduction in IBS severity score as early as 4 weeks [7].

Curcuma and Boswellia extracts, both formulated in sunflower lecithin (Phytosome™) and processed with a strong commitment to sustainability, have individually demonstrated beneficial effects on intestinal microbiota [8,9]. Moreover, a previous study [10] showed positive effects of a food-grade combination of *Curcuma longa* and *Boswellia serrata* on abdominal bloating and abdominal pain of subjects with IBS.

The present study aims to evaluate the effect of a lecithin formulation of Curcuma and Boswellia extracts on small bowel dysbiosis in IBS patients and to compare it with the effect of LFD.

## 2. Materials and Methods

This study was a randomized, double-blinded, placebo-controlled, parallel-group study with 30 days of clinical intervention. The two groups were randomly assigned to patients consecutively in chronological order of enrollment, according to the numbering from 1 to 49. The relative product packages provided by the production workshop were identified only with a number from 1 to 49, corresponding to the patient; the composition was only known to the manufacturing workshop. Participants were randomized to either the supplement (identified as the intervention group) or control arm (treated with a placebo and identified as the control group). Both groups followed a low-FODMAP diet (LFD). This study was carried out at the Department of Public Health of the University of Pavia, Italy. This study aimed to assess the efficacy and safety of CUBO in IBS subjects with dysbiosis of the small bowel. This study was carried out in agreement with the Declaration of Helsinki and the ICH Guidelines for Good Clinical Practice, following approval from the Independent Local Ethics Committee (Ethic code number: 0612/22052019). Written informed consent was acquired from all participants. This study was carried out from 3 September 2022 to 3 March 2023.

### 2.1. Population

Female and male adult subjects with a diagnosis of IBS and dysbiosis of the small bowel were potential candidates for this study. The inclusion criteria were as follows: 1. age ranging from 18 to 70 years; 2. diagnosis of moderate IBS according to Rome IV criteria [11,12]; 3. dysbiosis of the small bowel, defined by increased urinary indican values with normal values of urinary skatole [13,14]; and 4. evidence of abdominal bloating and abdominal pain.

Exclusion criteria were normal values of urinary indican or increased values of urinary skatole; subjects who were already on a low-FODMAP diet (LFD) or other dietary prescription, such as lactose or gluten-free diet in the last 6 months; insulin-dependent diabetes or seafood, nuts, or soy allergies; positive history of symptomatic diverticular disease, celiac disease, inflammatory bowel disease, or microscopic colitis; colonic, small bowel, or gallbladder surgery; severe vomiting or bloody diarrhea; liver disease (defined as altered values of liver function tests), severe renal disease (defined as serum creatinine > 1.5 mg/dL), or treatment with antibiotics, excluding those for topical use, within the last 3 months.

### 2.2. Supplementation and Concomitant Medications

Subjects included in the supplemented group received an LFD regimen together with CUBO™, a daily film-coated tablet containing a combination of 250 mg of *Curcuma longa* L. as sunflower lecithin-based formulation (Meriva™) and 125 mg *Boswellia serrata* extract as sunflower lecithin-based formulation (Casperome™) supplemented twice in a day for 30 days (provided by Indena S.p.A., Milan, Italy). The selection of this dosage was based on our previous experimental data with Boswellia and Curcuma phospholipids [10]. Before release, the film-coated tablets were tested for appearance, uniformity of mass, average mass, HPLC content of extracts of Boswellia and Curcuma, microbiological quality, and disintegration time. Supplementation compliance was calculated as the ratio between the number of returned tablets of supplement at the end of this study and the number of tablets corresponding to the expected intake during the treatment period. Subjects in the control group followed an LFD regimen without receiving any supplementation.

All subjects (intervention group and control group) were instructed by the same dietician to properly follow the LFD [15].

The concomitant use of other medications that could interfere with the results of this study was not allowed, including anxiolytics, analgesics (e.g., paracetamol), and spasmolytics (e.g., butylscopolaminium bromide).

### 2.3. Clinical Evaluation

Study visits were scheduled at baseline (day 0) and at day 30. A questionnaire was filled out by all participants on both visits on the following:

1. The score of abdominal bloating. Participants were asked whether they felt “bloating/uncomfortably full”. Four options could be chosen: none (symptom did not occur), mild (symptom occurred but did not interfere with usual activities), moderate (occurrence of symptom somewhat interfered with usual activities), or severe (occurrence of symptom resulted in an inability to perform usual activities) [16].
2. The score of the abdominal pain intensity. The score was defined using a validated visual analog scale score for pain (0 for “no pain” to 10 for “most severe pain”) [17,18].

The evaluation of small bowel dysbiosis was performed within 7 days before study inclusion and at the end of this study by the assessment of the indican and skatole in urine. Urinary values of indican (3-indoxyl sulfate) and skatole (3-methyl-indole) were evaluated through a colorimetric and high-performance liquid chromatographic method, respectively [19]. Urinary indican and skatole were considered normal when the levels were lower than 10 mg/L for indican and 10 µg/L for skatole [19].

According to Kruis and colleagues, at the end of this study, the global assessment of efficacy (GAE) was fulfilled by all subjects using a four-point scale (1 for “ineffective”, 2 for “moderately effective”—slight improvement of complaints, 3 for “effective”—marked improvement in symptoms, and 4 for “very effective”—as good as no symptoms) [20].

Vital signs were evaluated, and serum samples were collected during each of the two visits. Urine samples were collected on day 30. Laboratory tests were carried out and analyzed concerning the normal ranges. Participants were required to provide prompt information about any side effect, considered an adverse event, throughout this study.

### 2.4. Study Endpoints

The primary endpoint was the change in small bowel dysbiosis established by the decrease in urine indican values. Secondary endpoints included (1) the reduction in intensity of abdominal bloating, (2) the reduction in abdominal pain, and (3) the GAE as defined by all the participants at day 30. Safety endpoints were based on laboratory tests, vital signs, and the occurrence of adverse events.

### 2.5. Statistical Analysis

The sample size was estimated for the primary endpoint (urinary indican) using Zhao’s formula for the Wilcoxon–Mann–Whitney test. With an alpha of 0.05, a power of 0.90, an allocation rate of 1:1, and assuming that after supplementation, 40% of treated subjects had better scores (i.e., 0 or 1) in urinary indican compared with 10% of control subjects, the calculated total sample size was 49. Baseline data were shown as the mean values ± standard deviation of the mean (SD) unless otherwise indicated. The normal distribution of the variables was checked using the Shapiro–Wills test and using Q–Q graphs. Baseline differences in demographic and clinical characteristics between the intervention group and the control group were analyzed using independent *t*-tests.

The homogeneity of the variances was calculated using the Levene’s test. The one-factor covariance ANCOVA test was used to test the primary outcome (dysbiosis) and secondary outcomes as continuous variables to determine differences between the two cohorts. Mean and 95% CI were reported.

The same model was used to compare changes in the variables regarding the effects between the two cohorts: the effect was quantified by the intervention group minus controls adjusting for age and gender. The Statistical Package for the Social Sciences version 28 software was used to perform the statistical analysis (SPSS Inc., Chicago, IL, USA).

## 3. Results

**Figure 1:**
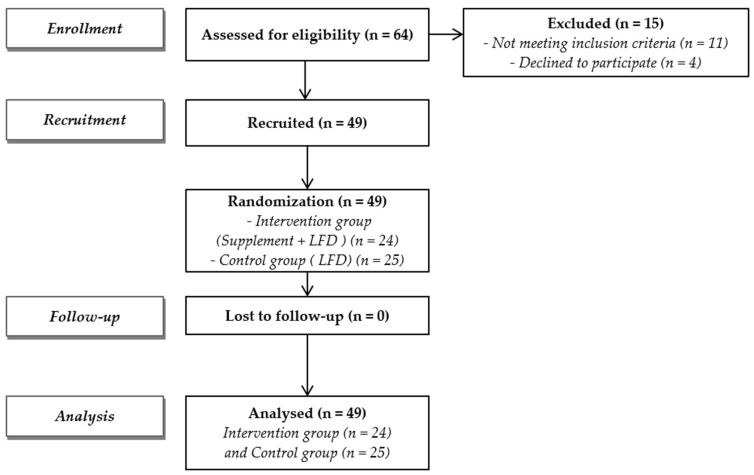
Flow diagram of this study.

**Figure 2:**
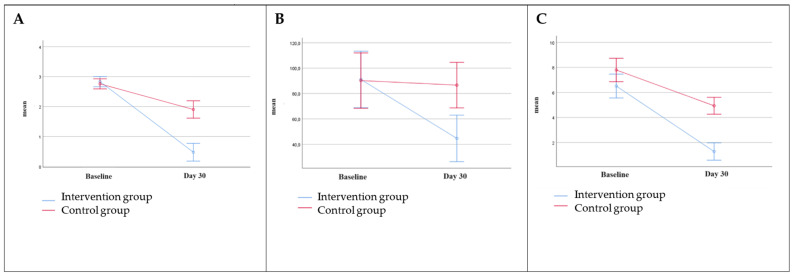
Correlation between groups on abdominal bloating, reduction in dysbiosis (urinary indican), and abdominal pain: (**A**) abdominal bloating; (**B**) urinary indican (mg/L); and (**C**) abdominal pain.

**Figure 3:**
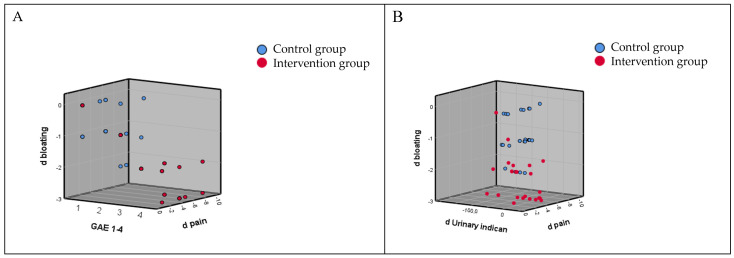
(**A**) The reduction and correlation between positive changes of abdominal bloating, abdominal pain, and GAE in the intervention group and control group; and (**B**) correlation between reduction in dysbiosis and positive changes in abdominal bloating and abdominal pain in the intervention group and control group.

Sixty-four subjects with IBS and dysbiosis of the small bowel were screened, and 49 (76.5%), 37 females and 12 males, with a mean (±SD) age of 43.9 (±14.4) years, were recruited (Figure 1). Eleven out of sixty-four subjects were excluded because they did not meet the inclusion criteria (six had a urinary indican value within normal limits, and five had urinary skatole values higher than normal limits), and four subjects declined to participate for personal reasons. Twenty-four out of forty-nine enrolled patients were randomly assigned to the intervention group, while the remaining twenty-five were included in the control group. (Figure 1). Table 1 reports the baseline variables of the two groups with the absence of statistically significant differences when the two groups were compared. Table 2 reports the pre–post intra-group data and the comparison between the two groups. The supplemented group shows a significant score reduction in the primary endpoint related to the urinary indican (*p* < 0.001), as well as for the secondary endpoints: abdominal bloating (*p* < 0.001) and abdominal pain (*p* < 0.001). On the contrary, the pre–post intra-group analysis of the control group did not evidence significant differences. The comparison between the two groups (net effect in intervention minus control subjects) showed that the changes differ significantly for urinary indican *p* < 0.001 (−42.88; 95% CI: −62.04 to −23.72), abdominal bloating *p* < 0.001 (−1.50; 95% CI: −1.93 to −1.07), and abdominal pain *p* < 0.001 (−2.37; 95% CI: −3.61 to −1.13) (Table 2). Figure 2 shows the correlation between groups on abdominal bloating, urinary indican, and abdominal pain. Table 3 shows the values of GAE measured at day 30. A statistically significant difference (*p* < 0.001) was observed when the two groups were compared. Very interestingly, almost 92% of CUBO™-supplemented subjects reported high efficacy (score 3 + 4) in respect of 12% of controls (score 3 + 4). Figure 3A evidences the correlation between positive changes in abdominal bloating, abdominal pain, and GAE in the intervention group and control group; Figure 3B shows the correlation between the reduction in dysbiosis and positive changes in abdominal bloating and abdominal pain in both the intervention and control groups.

## 4. Discussion

**Figure 4:**
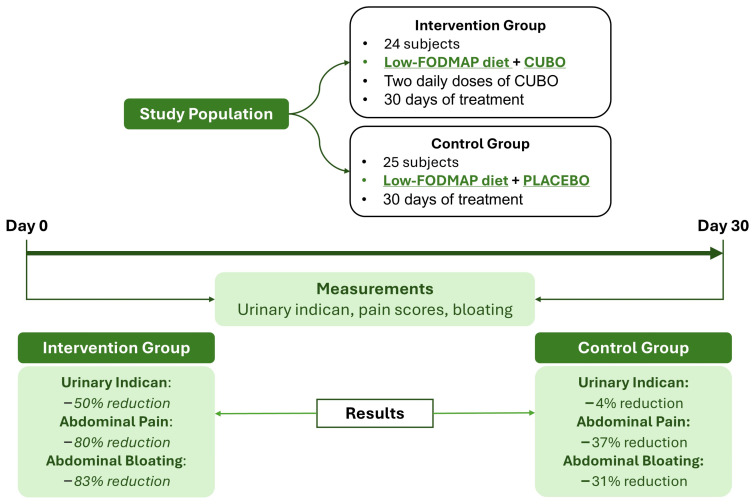
Lecithin-based delivery formulation of *Curcuma longa* and *Boswellia serrata* in IBS Patients.

This study shows that CUBO supplementation for 30 days favorably modifies small bowel dysbiosis in IBS subjects. This was the primary endpoint of this study. This is demonstrated by the significant reduction in indican values after supplementation with CUBO (intra-group analysis) and confirmed by the significant difference established when a comparison with the control group was performed (inter-group analysis), as shown in Figure 4.

It has been reported that Curcuma promotes the presence of beneficial bacterial strains in intestinal microbiota [8]. Interestingly, the interaction between natural curcuminoids, which are the metabolites of turmeric extracts, and microbiota is associated with two different phenomena, i.e., the favorable regulation of intestinal microflora by curcuminoids and the increased biotransformation of Curcuma extracts by gut microbiota [8,21]. Both events are crucial for the biological activity of *Curcuma longa* extracts [8,21].

In addition, rabbit diets supplemented with *Boswellia serrata* resin have been tested at different dosages to promote changes in the intestinal microbiota. These experiments showed marked changes in the microbial population in the cecum of rabbits supplemented with *Boswellia serrata*, with a significant reduction in total bacterial count and, specifically, a reduction in *Salmonella enteritidis* and *Escherichia coli*, compared to the untreated control group. These favorable results could be due to the high content of polyphenols of *Boswellia serrata* and the presence of boswellic acids, which have a significant antimicrobial activity [22]. The effect of the combination of Curcuma and Boswellia extracts on the microbiota could, at least in part, explain the positive effect of CUBO on dysbiosis in our study.

Small bowel dysbiosis, that is, small intestinal bacterial overgrowth (SIBO), is a frequent finding in IBS subjects with the presence in the small bowel of colon germs, including *Klebsiella*, *Escherichia coli*, or *Clostridia* [4,23]. The consequences of this overgrowth are represented by increased intestinal permeability, low-grade inflammation, and disruption of intestinal motility. Archaea species such as *Methanobrevibacter smithii* can also overgrow the small intestine. These bacteria produce methane gas in the small bowel, which may inhibit intestinal motility with possible delay of the intestinal transit. Increasing scientific evidence suggests a bidirectional communication between the gut microbiota and the enteric (ENS: enteral nervous system) and central nervous system (CNS), referred to as the “brain–gut–microbiome axis” [3]. This close communication assumes that intestinal bacteria could influence human brain activity indirectly or directly via the ENS. Whether the brain function is positively or negatively influenced depends on the microbiota composition of symbiotic or pathogenic bacteria [3]. In small bowel dysbiosis, the overgrowth of pathogens leads to an increased release of harmful rather than beneficial bacterial metabolites that may be involved in the pathogenesis of IBS. Therefore, the control and modulation of small bowel dysbiosis appears of great importance in the clinical management of IBS, and the results of this study pave the way for a new beneficial approach in this field. Future and more analytical research dedicated to this topic is required.

Few studies have attempted to characterize the small intestinal microbiota, primarily due to the difficulty in accessing the small intestine and lower microbial density, making it difficult to obtain sufficient bacterial DNA [24]. Elevated levels of indican are considered an indicator of the overgrowth of anaerobic bacteria in the small intestine, impaired protein digestion, or excess protein (tryptophan) ingestion. Therefore, it is a useful tool in SIBO diagnosis. This test has been used in this study, even though the gold standard for diagnosing small intestinal bacterial overgrowth uses a jejunum aspirate and culture. However, due to the invasiveness of the test, breath tests with glucose or lactulose or the urine indican test are more commonly used [25,26,27].

The low-FODMAP diet is considered a standard of care for IBS, specifically for abdominal bloating in IBS subjects. In our experience, LFD, followed by the controls, showed a lower efficacy in small bowel dysbiosis when compared with IBS subjects supplemented with CUBO and LFD. This finding confirms the relevant role of the association of Curcuma and Boswellia phospholipids in achieving the primary endpoint of this study, i.e., the decrease in intestinal dysbiosis in IBS subjects. Supplementation with CUBO has also been demonstrated effective for the secondary endpoints, i.e., bloating and abdominal pain. Both these targets have been achieved with a clear significant reduction in the bloating score and the pain score only in the intervention group supplemented with CUBO and with evidence of a significant difference when supplemented subjects and controls were compared. These data confirm our previous study with higher doses of curcumin and a different formulation of *Curcuma longa* and *Boswellia serrata* [10].

The benefits of CUBO were also confirmed by the evaluation of the global assessment of efficacy (GAE). At the end of this study, a significantly higher number of subjects in the supplemented group reported a marked improvement in symptoms (GAE score 3) and a complete absence of symptoms (GAE score 4).

The mathematical analysis of our data using the dispersion test shows a significant correlation between the reduction in dysbiosis and the reduction in abdominal bloating and pain only in the supplemented group, thus showing that intestinal dysbiosis plays a crucial role in the appearance of symptoms in IBS subjects (Figure 3B). Moreover (Figure 3A), a relevant correlation between the reduction in abdominal bloating and abdominal pain with the highest scores of GAE was observed only in the CUBO-supplemented subjects, while in the control group, it was not found. Together, these data underline the tight correlation between intestinal dysbiosis, abdominal pain, and bloating and the patient’s perception of treatment efficacy.

The evaluation of the sex–gender differences showed that male subjects supplemented with CUBO and LFD had a 20% better result when compared with females. However, conclusions of a recent systematic review (van Kessel et al., 2021 [28]) reported that various types of IBS treatment, and in particular the use of alosetron, a serotonin antagonist, the treatment with inoculant and crofelemer, and the combination of cognitive behavioral therapy with pharmacological treatment, show significantly different effects in the two sexes, with benefits for women, both in the reduction in overall IBS symptoms and in the number of pain-free days. This review was based on a limited number of existing studies, and other reviews [29,30] underlined the complexity of factors (i.e., hormones, pain perception, stress, microbiota) that can affect sex–gender differences observed among different studies. Therefore, we suggest that more attention must be paid in the future to gender as an influencing, maybe even determining factor, on the potential effectiveness of care for IBS subjects.

Phytosome technology is a food-grade formulation used for natural plant products to obtain a solid dispersion by mixing a carrier such as sunflower lecithin (phytosomes) and the active ingredients of the product to increase the area of exposure of natural molecules or extracts to the gastrointestinal layer. Bresciani et al. showed that the phytosome formulation significantly influenced the biotransformation of curcuminoids, with increased production of curcuminoid catabolites using the fermentation of lecithin-curcuminoids by the fecal human microbiota [21]. In our study, both the extracts were formulated in phytosome, and this could have favored the positive clinical outcome due to a potential increase in the exposure area of botanical extracts activity and an increase in the extract’s active catabolites.

The dosage of the final formulation of Curcuma and Boswellia phytosomes used in this study was lower as compared with previous studies performed in patients with diverticular disease as well as in IBS subjects [31], suggesting that the phytosome technology might have favored the reduction in the efficacious dosage of CUBO, maintaining its benefits. Although many human studies confirmed the safety of curcumin even at high doses of 12 g/day, high doses of herbal compounds in long-term use have been shown to cause digestive problems or other acute toxicities [32]. For this reason, the use of a low dose of curcumin, as applied in this study, appears of considerable importance.

The limitations of this study could be ascribed to the small number of recruited subjects and the absence of a placebo supplementation. It is well known that the placebo effect in IBS patients is high, and specific studies with a placebo group should be conducted in the future [33]. An additional limitation of this study is the test chosen for the evaluation of small bowel dysbiosis. This is a surrogate test, and these results should be confirmed by other studies with measurement of hydrogen and methane in breath samples [34,35].

For these reasons, additional studies are needed to confirm our observations, and potential areas of future research could be the involvement of large samples of different types of IBS patients and the evaluation of the long-term effects of CUBO on IBS symptoms and gut health.

## 5. Conclusions

In conclusion, irritable bowel syndrome is a chronic gastrointestinal disorder in which abdominal pain or discomfort is associated with defecation or changes in bowel habits and with evidence of small bowel dysbiosis. Its multifactorial pathophysiology leads to a variety of available treatments, mainly aimed at controlling symptoms. The management of IBS patients could be optimized by individualized strategies, including non-pharmaceutical approaches [10].

CUBO, the cooperative combination of natural curcuminoids formulated in phytosome and Boswellia phytosome, has proven to have significant capabilities in improving IBS subjects’ gut health. When associated with a low-FODMAP diet, it decreases intestinal dysbiosis in IBS subjects and contributes to a reduction in abdominal bloating and pain to a greater extent compared with a low-FODMAP diet and placebo. CUBO acts on intestinal health both objectively—as proven by the significant decrease in urinary indican—and subjectively, with over 92% of subjects claiming their conditions improved noticeably. Thanks to the combined action of two botanical ingredients with remarkable properties, CUBO opens up a new dimension of gut wellbeing, simultaneously carrying out different actions for the health of the intestine while also providing relief from bloating and abdominal pain, with a potential positive modulation of the gut microbiota.

Additional research is needed to confirm these findings, focusing also on different subtypes of IBS and the gender of patients with IBS.

## Figures and Tables

**Table 1:** Descriptive statistics of the participant characteristics at baseline and statistical comparison between the intervention and control groups.

Variable	Control Group (n = 25) 20 F, 5 M	Intervention Group (n = 24) 17 F, 7 M	Total (n = 49) 37 F, 12 M	*p*-Value Between Groups
	Mean (SD)			
Age (years)	41.76 (14.23)	46.25 (14.59)	43.96 (14.43)	0.281
Abdominal bloating (score)	2.76 (0.43)	2.83 (0.38)	2.80 (0.40)	0.534
Urinary indican (mg/L)	89.10 (57.29)	92.22 (50.03)	90.63 (53.32)	0.841
Pain (score)	7.76 (2.35)	6.54 (2.55)	7.16 (2.35)	0.070

**Table 2:** Intra- and inter-group changes from baseline (day 0) to the end of this study (day 30) for the investigated variables.

Variable	Controls Mean Difference Changes (95% CI)	Controls Mean Difference Change in Percentage (%)	Intervention Group Mean Difference Changes (95% CI)	Intervention Group Mean Difference Change in Percentage (%)	Between Groups Mean Difference Changes (Supplemented Minus Control Effect) (95% CI)	Between Groups Mean Difference % (Supplemented Minus Control Effect)	*p*-Value Between Groups
Urinary indican	−3.58 (−16.89, 9.72)	−4	**−46.47** **(−60.06; −32.88)**	**−50**	**−42.88** **(−62.04, −23.72)**	**−46**	*p* < 0.001
Abdominal bloating	−0.85 (−1.71, 0.01)	−31	**−2.35** **(−2.66, −2.05)**	**−83**	**−1.50** **(−1.93, −1.07)**	**−52**	*p* < 0.001
Abdominal pain	−2,86 (−3,72; 1,99)	−37	**−5.23** **(−6.115; −0.347)**	**−80**	**−2.37** **(−3.619; −1.13)**	**−43**	*p* < 0.001

In bold data statistically significant at *p* < 0.05 level.

**Table 3:** Frequencies of GAE (global assessment of efficacy) at the end of this study.

GAE	Intervention Group	Control Group	Total	*p*-Value
Score 1 (n)	1	14	15	*p* < 0.001
%	4.2	56.0	30.6	
Score 2 (n)	1	8	9	
%	4.2	32.0	18.4	
Score 3 (n)	8	3	11	
%	33.3	12.0	22.4	
Score 4 (n)	14	0	14	
%	58.3	0.0	28.6	

## Data Availability

The data presented in this study are available on request from the first author of the study (Attilio Giacosa, coordinator of the study).
